# Non-Invasive Detection of Respiration and Heart Rate with a Vehicle Seat Sensor

**DOI:** 10.3390/s18051463

**Published:** 2018-05-08

**Authors:** Grace Wusk, Hampton Gabler

**Affiliations:** Department of Biomedical Engineering and Mechanics, Virginia Tech, 325 Stanger Street, Blacksburg, VA 24061, USA; gabler@vt.edu

**Keywords:** advanced automatic collision notifications, seat sensor, respiration rate, heart rate, ballistocardiography

## Abstract

This study demonstrates the feasibility of using a seat sensor designed for occupant classification from a production passenger vehicle to measure an occupant’s respiration rate (RR) and heart rate (HR) in a laboratory setting. Relaying occupant vital signs after a crash could improve emergency response by adding a direct measure of the occupant state to an Advanced Automatic Collision Notification (AACN) system. Data was collected from eleven participants with body weights ranging from 42 to 91 kg using a Ford Mustang passenger seat and seat sensor. Using a ballistocardiography (BCG) approach, the data was processed by time domain filtering and frequency domain analysis using the fast Fourier transform to yield RR and HR in a 1-min sliding window. Resting rates over the 30-min data collection and continuous RR and HR signals were compared to laboratory physiological instruments using the Bland-Altman approach. Differences between the seat sensor and reference sensor were within 5 breaths per minute for resting RR and within 15 beats per minute for resting HR. The time series comparisons for RR and HR were promising with the frequency analysis technique outperforming the peak detection technique. However, future work is necessary for more accurate and reliable real-time monitoring of RR and HR outside the laboratory setting.

## 1. Introduction

Advanced Automatic Collision Notification (AACN) systems have great potential for reducing mortality risk in car crashes. AACN systems however, rely exclusively on vehicle-based measures of crash severity from which occupant crash response can only be estimated [1,2,3,4]. Non-invasive physiological monitoring of an occupant could prove extremely valuable in improving occupant safety for post-crash emergency response. Relaying vital signs of the occupants after a crash to the first responders, prior to arrival at the crash scene, could help prepare the appropriate response for transport as well as medical triage. Injury predictions could also better prepare emergency room doctors for treatment of incoming crash victims. The purpose of this study was to assess the feasibility of seat sensors in production passenger vehicles to not only classify an occupant according to weight, but to also monitor occupant respiration rate (RR) and heart rate (HR). 

We arrived at the proposed physiological monitoring technique after assessing a number of requirements. First, the system must be low cost for any practical application. Second, the sensor must be non-invasive and require no additional calibration or effort from the occupant. Ideally, as Weiser explains, a ubiquitous system should disappear into the background and be indistinguishable and fundamentally integrated into everyday life [5]. Third, the sensor must be able to physiologically monitor individuals with various weights and resting rates. Finally, the sensor must work in both light and dark, day and night, as long as the vehicle is in use.

There are a variety of off-body sensing techniques that can be used to detect RR and HR. A past study integrated three sensing systems into driver and passenger car seats to detect vital signs in a stationary and moving vehicle. The measurement techniques included capacitive electrocardiogram (ECG) monitoring, mechanical movement analysis, and inductive impedance monitoring, none of which require electroconductive contact to the human body [6]. For the capacitive ECG and inductive impedance monitoring, electrodes and coils were integrated into the backrests of car seats. The last technique used a quasi-piezoelectric force transducer to measure small body movements through the seat. The mechanical aspects of heart activity through blood movement can be measured through a technique referred to as ballistocardiography (BCG) [7,8]. This technique can also be applied to larger body movements such as breathing. Approaches using electromechanical film sensors and accelerometers have been tested for displacement and acceleration derived BCG, respectively, in moving systems such as a wheelchair [9]. Separating motion vibrations from the small cardiac movements is a challenge. However, adaptive cancellation of vibrations may help improve BCG signals [10]. For electromechanical films, sensitivity of the pressure sensors may also depend on the static load and the distribution of weight while sitting [11]. For example, weight may shift between the feet, arms, back, and behind while sitting, which would affect the BCG signal. Wearable BCG systems have also been explored and may benefit from a multi-sensor system to separate useful information from the noise of normal ambulation [12]. Using various pressure sensors to detect slight fluctuations in body movements, from breathing or pulsing, is also common in sleep tracking technologies [13,14]. Other sensors on the market, such as the Plessey Electric Potential Integrated Circuit (EPIC) sensor, have been tested in contact and non-contact modes to measure bio-electric signals and movement from disruptions in electric fields. These integrated sensors have been used to monitor respiration and heart rates and may be suitable for automotive applications [15]. Low-power radar sensors have also been proposed for contactless heart and lung monitoring [16]. Finally, extraction of occupant vital signs may be performed through video image processing [17,18]. However, the image processing techniques, as well as the other non-contact sensing methods discussed, would require the installation of additional hardware into a vehicle. 

In contrast, the proposed technique utilizes seat sensors that automakers have manufactured and integrated for years into vehicles with advanced airbags. Advanced passenger airbag systems require automakers to install occupant-sensing systems, such as seat sensors, to classify occupants [19]. These occupant classification systems may take the form of pressure-sensing mats or bladders under the seat cushions or as weight strain gauges integrated into the seat tracks. Using a BCG approach, we hypothesize that a vehicle seat sensor can detect RR and HR, offering a non-invasive approach to monitor occupants after a crash and to better predict injury severity. The proposed solution meets our criteria for a ubiquitous physiological monitoring technique to inform post-crash response and complement AACN vehicle-based information, while leveraging existing technology. 

## 2. Materials and Methods

**Figure 1:**
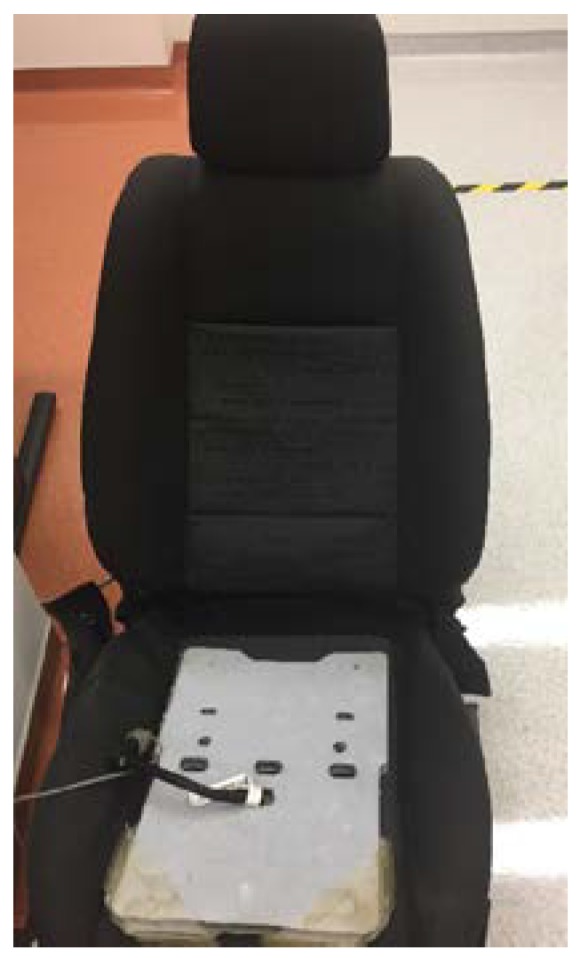
Ford Mustang front passenger seat and seat sensor.

The seat sensor used in this study was retrieved from a Ford Mustang front passenger seat and connected to a cDAQ-9172 data acquisition system (National Instruments, Austin, TX, USA). The sensor consists of a fluid filled bladder connected to a solid-state pressure transducer. Benchmark respiration and pulse measurements were collected with the Neulog Respiration Monitor Belt Logger Sensor and the Neulog Heart Rate and Pulse Logger Sensor (Neulog, Rochester, NY, USA). All of the data were sampled at 10 Hz. A Nyquist frequency of 10 Hz would be sufficient for a maximum signal frequency of 5 Hz, or 300 beats per minute. The seat and seat sensor used in the study are shown in Figure 1. The protocol was approved by the Virginia Tech Institutional Review Board (IRB) #17-018. Data samples were collected from eleven participants, three male and eight females. All participants were over the age of 18. Self-reported body weights ranged from 42 to 91 kg as shown in Table 1. Each participant sat on the seat for 30 min while seat sensor data and reference data were collected. During data collection, the seat sensor was placed under the foam cushion of the seat, as it would be in a vehicle. 

### 2.1. Respiration Rate (RR)

To extract the RR, the raw voltage output signal from the seat sensor was first filtered using a fourth-order Butterworth band-pass filter with cutoff frequencies of 0.16 Hz and 0.66 Hz. The cutoff frequencies were derived from physiological bounds of respiration rate, with normal resting RR for an adult ranging from 10 to 40 breaths per minute (brpm). We targeted no more than a 10% difference between the seat sensor RR and the Neulog RR, using the upper range of RR. This defines a target difference of 4-brpm for RR. 

**Figure 2:**
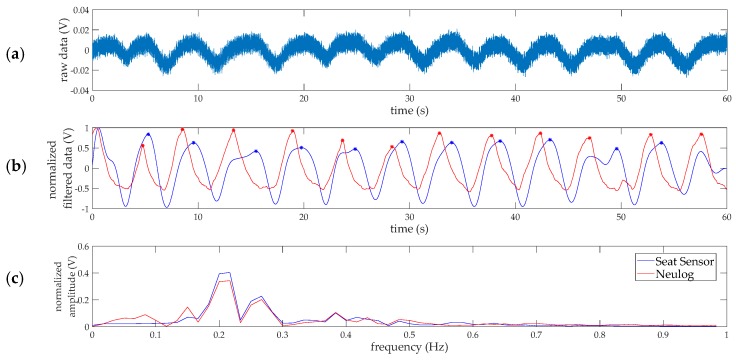
Sample respiration rate data processing and measurements for 1-min of data collection at 100 Hz. (**a**) Raw voltage output from the seat sensor; (**b**) the filtered breathing signal is shown in blue and the analog benchmark data from the Neulog respiration belt is shown in red. The signals are normalized and each peak, indicated by the stars, represents one breath; (**c**) the power spectrum for the seat sensor and benchmark data, showing the FFT frequency components composing the normalized signals.

Next, two methods were explored to calculate the brpm over time using the filtered respiration data. The data was processed within a 1-min sliding window for the 30 min of data collection per participant. The first technique was to count the peaks in the analog signal and convert to brpm. Peak detection was performed in MATLAB. Only peaks with a peak prominence of at least 90% of the average peak prominence for the 1-min windowed signal were included in the count. This thresholding strategy helped objectively ignore small fluctuations in the filtered signals. The second technique was to quantify the frequency content of the pressure signal using the fast Fourier transform (FFT) and compute RR based on the highest amplitude frequency from the respiration filtered data. Example data sampled at 100 Hz is shown in Figure 2 to illustrate the steps involved in data processing. The reference respiration signal (in arbitrary normalized units) from the Neulog respiration belt is overlaid in red on the filtered seat sensor data for comparison. For the example data in Figure 2 using the peak detection technique, the RR is 11 brpm and 12 brpm for the seat sensor and Neulog sensor, respectively. Using frequency analysis, the rate is 13 brpm for both measurement devices.

### 2.2. Heart Rate (HR)

The raw data was filtered for the HR calculation using a fourth-order Butterworth band-pass filter with cutoff frequencies of 0.83 Hz and 2.5 Hz. The cutoff frequencies would be sufficient for adults with a heart rate above 50 beats per minute (bpm) and below 150 bpm. The lower cutoff frequency of 0.83 Hz also served to remove the respiration signal from the analog data. We targeted no more than a 10% difference between the seat sensor HR and the Neulog HR, using the upper range of HR. This defines a target difference of 15-bpm for HR.

**Figure 3:**
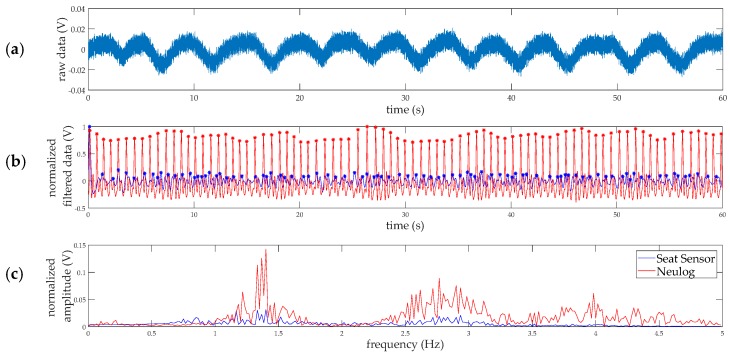
Sample heart rate data processing and measurements for 1-min of data collection at 100 Hz. (**a**) Raw voltage output from the seat sensor; (**b**) the filtered pulse signal is shown in blue and the analog benchmark data from the Neulog pulse monitor is shown in red. The signals are normalized and each peak, indicated by the stars, represents one breath; (**c**) the power spectrum for the seat sensor and benchmark data, showing the FFT frequency components composing the normalized signals.

Like the respiration rate, the HR was calculated in two ways to compare to the reference measurement using a 1-min sliding window. The heart rate was computed by counting peaks and by fast Fourier transform frequency analysis of the filtered seat sensor signal as shown in Figure 3. For the FFT analysis, the highest amplitude frequency determined the HR. The reference pulse signal (in arbitrary normalized units) from the Neulog pulse sensor is overlaid in red on the filtered seat sensor data for comparison. For the example in Figure 3, the peak detection method counted 91 bpm for the seat sensor and 83 bpm for the Neulog sensor. The frequency analysis resulted in 84 bpm for both measurement devices. The second harmonic seen in Figure 3c, around 2.8 Hz, is most likely from the secondary upstroke in the Blood Volume Pulse (BVP) signal. This smaller amplitude upstroke represents the interaction of the initial pressure wave from the heart and the reflected pressure wave from the body [20]. The “reflected” waves are visible in the analog signals in Figure 3b.

Data was collected for 30 min for each of the eleven participants. Each second, the previous 60 s of analog data, such as the raw data shown in Figure 2a and Figure 3a, was processed using the peak detection and FFT frequency analysis techniques to yield a RR and a HR for that time point. Data points for every second provided high temporal resolution and the 1-min sliding window provided sufficient time for the FFT frequency analysis. The respiration and heart rate time courses from the seat sensor were compared to the reference measurements. For this study, reference RR was calculated from the Neulog analog signal using the corresponding technique (peak detection or frequency analysis) whereas reference HR was directly output from the Neulog device in bpm. The Neulog device was not able to output the RR in brpm. The participants sat and relaxed during the 30 min of data collection, therefore, the mean RR and HR over the 30 min were considered the participants’ resting rates. Bland-Altman plots were used to compare the resting RR and resting HR from the two measurement devices. Bland-Altman plots show the relationship between inter-device recording differences and are a common method of measurement comparison for continuous variables [21,22]. As shown in a past study comparing Jawbone and Fitbit fitness tracking devices, the Bland-Altman plots typically show the differences between devices over mean values [23]. Simple linear regression models were run in R, regressing the seat sensor resting rates on the reference Neulog resting rates, to calculate RMSE and R^2^ values. 

The RR and HR measures from the two devices were also compared at each individual time point to evaluate the seat sensor performance at a higher resolution of time. To assess the reliability of the measurements over the range of occupant weights and resting RR and HR, the data was visualized with Bland-Altman style plots. Ideally, the differences between seat sensor readings and the reference readings are not only minimal, but also independent of body weight and resting rate. The measurement differences (Neulog values subtracted from seat sensor values) were plotted against participant weight and resting rates. Using the pooled differences from all participants and time points, the mean and standard deviation (SD) of the differences were calculated for both methods, peak detection (Peaks) and frequency analysis (FFT), for RR and HR. Levels of agreement were calculated for the differences using a 95% confidence interval (CI). In comparison to the models using resting rates, simple linear regression models were also created using the RR and HR time series data to calculate RMSE and R^2^ values. 

## 3. Results

**Figure 4:**
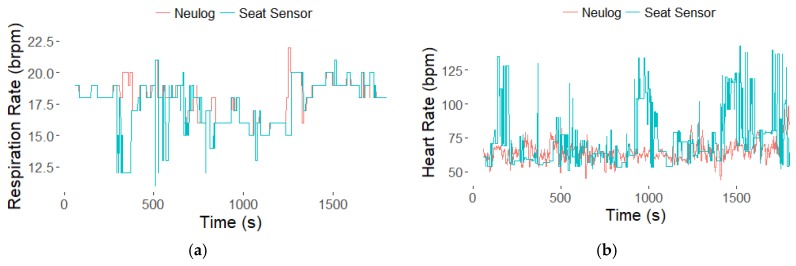
Example data from Participant 2 (P2). The respiration (**a**) and heart (**b**) rate time courses from the seat sensor using the frequency analysis (FFT) technique.

Figure 4 shows example RR and HR time courses from the frequency analysis data processing technique. While the RR and HR calculated from the seat sensor fluctuate more than the reference rates, they generally agree with the expected values. In Figure 4, there is a decrease in RR around 1000 s, which the seat sensor is able to detect. 

**Figure 5:**
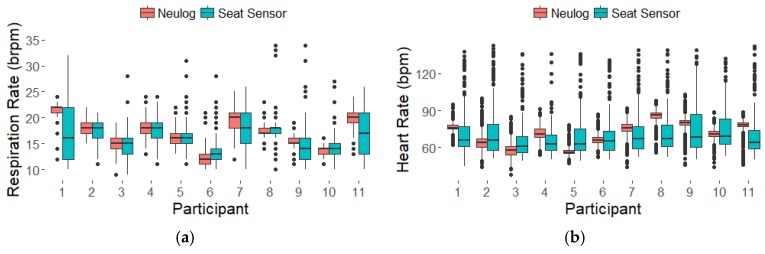
Boxplots of respiration (**a**) and heart (**b**) rates derived from the frequency analysis technique for all eleven participants over 30-min data collection.

**Figure 6:**
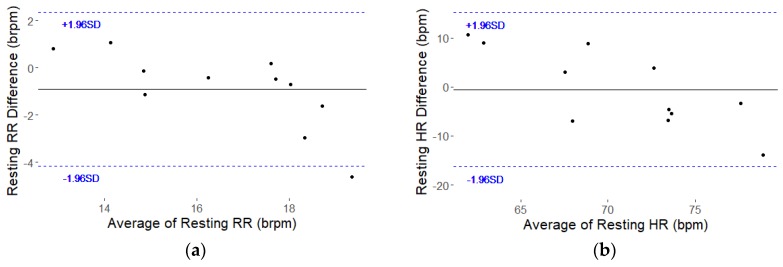
Bland-Altman plots for resting RR (**a**) and resting HR (**b**). Difference between seat sensor and Neulog over the average of the two measures. Solid black line shows mean differences and dashed blue lines show 1.96 standard deviation bounds.

The boxplots in Figure 5 summarize the data across all eleven participants, showing larger spread in the seat sensor results but comparable means over the 30 min. Only results from the frequency analysis are shown. The mean RR and HR over the 30 min were considered the participants’ resting rates. The resting rates for the eleven participants are listed in Table 2 and correspond with the boxplots in Figure 5. The differences between the seat sensor and Neulog for resting RR were below our 4-brpm target for 10 of the 11 participants as shown in Table 2. For resting HR, the differences for all the participants were less than our 15-bpm target as shown in Table 2. The Bland-Altman plots for resting RR and resting HR are shown in Figure 6. The plots show descending trends in the resting RR and HR differences which were tested by fitting lines to the data. The slope of the fitted line for RR was −0.57 (*p* = 0.0111) with R^2^ = 0.53. For HR the slope was −1.16 (*p* = 0.0039) with R^2^ = 0.62. These significant slopes indicate a systematic error, with positive differences for lower average values and negative differences for higher average values as shown in Figure 6. Regressing the resting RR from the seat sensor FFT on the resting RR from the Neulog resulted in a RMSE of 1.6 and a R^2^ of 0.72. Regressing the resting HR from the seat sensor FFT on the resting HR from the Neulog resulted in a RMSE of 8.4 and a R^2^ of 0.21.

**Figure 7:**
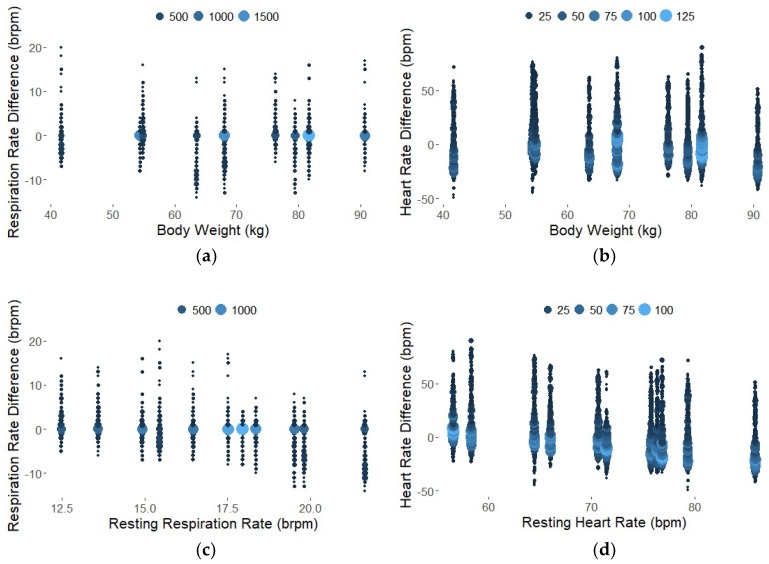
Bland-Altman plots to show reliability over weight (**a**) and (**b**) and over resting rates derived from the mean values over 30 min (**c**) and (**d**). Each data point represents the difference between the RR or HR calculated from the seat sensor compared to the Neulog for every time point and every participant. The size and color represent the density of overlapping points.

The Bland-Altman style plots in Figure 7 show the differences between the measurement devices across participant weight and resting rates for all time points, pooled from all participants. Each participant trial consisted of 1741 data points, RR and HR calculated every second from time 1 min to 30 min. The size and color of the points represent the number of data points in a single location to illustrate the high density of points around the 0-difference line. The differences are relatively consistent across body weight and resting rates for RR and HR. Summary statistics for the differences between the measurement devices and the simple linear regression models for all data points are provided in Table 3. As shown in Table 3, the frequency analysis technique resulted in lower difference means, or bias, but higher difference standard deviations. Interpreting the confidence interval, we expect the difference in respiration rate between the seat sensor and the Neulog (reference) sensor to be between −7.5 brpm and 5.7 brpm for 95% of future FFT measurements. These values exceed our target value of a 4-brpm difference between seat sensor RR and Neulog RR, however the results are improved by averaging measurements over time. The bias, or difference mean, for RR with the seat sensor was less than one brpm over the entire 30-min period. For heart rate, 1.96 standard deviations on either side of the mean results in a difference range of −39 bpm and 38 bpm, which is greater than our 15-bpm target. Averaging measurements over time improves results as the overall bias of the seat sensor for HR with FFT was less than one beat per minute for the 30-min data collection. 

The Root Mean Square Error (RMSE) values indicate the absolute fit of the linear model, with the stipulation that the model slope may not be exactly one. The benefit of RMSE is that the values are in the appropriate units. In this case, 2.8 brpm and 9.9 bpm of variation in the model may be acceptable. The small R^2^ values express the variability of the seat sensor RR and HR when computed every second using a 1-min sliding window. While we see a high density of data points on the 0-difference line in the plots in Figure 7, there are a number of outliers, especially for the heart rate data. However, when deciding whether the seat sensor RR and HR values are sufficiently close and therefore suitable predictors of the Neulog values, it is important to remember than R^2^ is a relative measure of fit.

## 4. Discussion

Extracting RR and HR from an occupant classification system presents a challenge due to the attenuation of the signals traveling from the lungs and heart to the seat sensor. In addition, differences in respiration and heart rate signal level, including frequency and amplitude, make the detection task difficult. The results of this study show the feasibility of using a production vehicle passenger seat sensor to measure physiological responses such as RR and HR for a variety of occupant weights and resting rates. The resting, or average, RR and HR values were comparable between the seat sensor and Neulog devices. Additional analysis was conducted to compare individual time points throughout the 30-min data collection. The primary analysis used to assess continuous differences between the Neulog and seat sensor devices was the Bland-Altman approach. Linear regression and correlation statistics were also reported, although they have been criticized for use in measurement comparison studies [22]. As expected, the RMSE values were higher and R^2^ values were lower for the model with all the data points compared to the model with resting rates derived from 30 min of data. This suggests that averaging the data over longer periods improves agreement with the reference values. Future work is needed to improve temporal resolution of RR and HR detection for continuous, real-time applications.

In the future, enhancing the peak detection algorithms could improve the calculation of RR and HR from total analog peaks. Empirical testing of different prominence threshold values could improve peak detection. In this study, the data collection consisted of normal, resting state, sustained breathing, with minor exceptions as shown in Figure 4a, with a visible decrease in RR, perhaps because of drowsiness. While averaging values of RR and HR over a 30-min data collection is reasonable for a normal, resting state, an occupant’s physiology may change drastically in minutes. Our first goal was to confirm that we could capture steady state response. Our follow-on goal will be to measure transient response by assessing RR and HR every second using a 1-min period of data. Additional work is required to evaluate the accuracy of detecting transient responses. The size of the sliding window, currently 1 min, would be an interesting variable to test in future experimentation. The FFT frequency analysis would benefit from a larger time window at the cost of delayed results. 

Detecting more drastic and transient changes in the respiration and heart rate in a running vehicle would be necessary for future applications of real-time monitoring during driving. Non-invasive measurement of occupant RR and HR could be useful for future work on psychophysiological classification of driver’s state [24]. Complementary to vehicle-based assessment which uses vehicle data such as steering and speed variability to assess the driver; physiological monitoring could provide a direct measure of the driver state. Combining basic physiological measurements could help automatically detect driver physical impairment such as fatigue. However, this application would require seat weight sensors in the driver seats while the current U.S. regulatory requirement is for passenger seats only. The ability to predict a driver’s state would be extremely beneficial in the design of future driver assistance systems and active safety technologies.

A major limitation of this study was the removal of the test seat and seat sensor from a vehicle. As shown in a previous study, an engaged engine and/or driving may lead to a low signal to noise ratio in a BCG signal from a seat sensor [6]. While movements and vibrations during driving could affect the detection of RR and HR, the goal of this study was to show feasibility and test the sensitivity of an occupant classification system from a production vehicle. Additionally, when considering post-crash response, a moving vehicle may not be a concern to an occupant-sensing system as the vehicle may be stopped. Another limitation was that testing did not include out-of-position occupants. The assumption is that the occupants are fully seated during sensing, which may imply seat belt use and exclude cases such as rollover crashes in which the vehicle remains upside down post-crash. Depending on the injury and possible blood loss, tachycardia or bradycardia may be observed in an occupant post-crash [25]. Abnormally high or low HR could approach the bounds of the current model with cutoffs at 50 bpm and 150 bpm. Likewise, RR is variable depending on injury. The current model is designed to capture RR from 10 to 40 brpm. We also assumed the performance of the occupant classification system would not be affected by a crash. The study could benefit from a larger and even more diverse dataset of human participants. Ideally, the seat sensor would be able to detect RR and HR in both a 5th percentile female, which would be around 50 kg, and a 95th percentile male, which would be around 125 kg, in the United States [26]. The participant pool used in this study covers the lower end of body weight requirements, but data from a participant heavier than 91 kg is lacking in this study. Finally, while the Federal Motor Vehicle Safety Standard (FMVSS) Number 208 mandates that modern vehicles have an occupant classification system for advanced passenger airbag systems, every manufacturer’s technique and technology may be different [19]. To expand this study, it would be valuable to test seat sensors from various vehicle makes and models.

## 5. Conclusions

This study provides promising results for using a seat sensor from a production passenger vehicle to measure occupant respiration and heart rate. Two calculations of RR and HR, peak detection and frequency analysis, were compared to reference measurements. Differences between the seat sensor and Neulog sensor for resting RR and HR were mostly within our 4-brpm and 15-bpm targets, respectively. Ideally, the system would also be able to assess RR and HR over time. Comparing the seat sensor to the reference sensor for all time points, the frequency analysis technique resulted in a smaller mean difference, or bias, than the peak detection technique. This data provides initial validation of the non-invasive seat sensor in a laboratory setting. Future testing in a running vehicle is necessary for applications of occupant monitoring. Non-obtrusive, direct measures of the occupant using hardware already installed in all modern vehicles could have a large impact on occupant vehicle safety, in terms of both post-crash emergency response and crash avoidance technologies.

## Figures and Tables

**Table 1:** Participants.

	P1	P2	P3	P4	P5	P6	P7	P8	P9	P10	P11
Gender	F	F	M	M	F	F	F	F	F	M	F
Weight (kg)	64	54	82	82	68	55	79	91	42	76	68

**Table 2:** Resting RR and HR Derived from Seat and Neulog Sensors for Eleven Participants

		P1	P2	P3	P4	P5	P6	P7	P8	P9	P10	P11
RR	Seat (brpm)	17	17	15	18	16	13	18	18	14	15	17
	Neulog (brpm)	22	18	15	18	16	12	20	18	15	14	20
	Difference (brpm)	−5	−1	0	0	0	1	−2	0	−1	1	−3
HR	Seat (bpm)	71	73	67	65	67	69	71	72	76	75	70
	Neulog (bpm)	76	64	58	71	57	66	76	86	79	71	77
	Difference (bpm)	−5	9	9	−6	10	3	−5	−14	−3	4	−7

**Table 3:** Summary Statistics for the Peak Detection and Frequency Analysis Techniques for RR and HR using all Time Points across all Participants.

Signal	Units	Analysis Method	Difference Mean	Difference SD	Difference 95% CI	RMSE	R^2^
RR	brpm	Peaks	−2.5	2.9	(−8.3; 3.3)	2.5	0.16
		FFT	−0.91	3.3	(−7.5; 5.7)	2.8	0.22
HR	bpm	Peaks	−23	13	(−48; 2.9)	9.9	0.0019
		FFT	−0.50	19	(−39; 38)	9.9	0.0043
